# Brucea javanica oil inhibited the proliferation, migration, and invasion of oral squamous carcinoma by regulated the MTFR2 pathway

**DOI:** 10.3389/fonc.2024.1477293

**Published:** 2025-01-13

**Authors:** Yihan Lai, Mingkang Li, Juan Zhan, Lin Jiang, Yuan Wu, Zhiyi Fang, Jianhan Zhou, Yujie Ma, Yisen Shao, Wei Wang

**Affiliations:** ^1^ Department of Oral and Maxillofacial Surgery, Affiliated Hospital of Jiangxi University of Chinese Medicine, Nanchang, Jiangxi, China; ^2^ Jiangxi University of Chinese Medicine, Nanchang, Jiangxi, China; ^3^ Key Laboratory of Oral Diseases of Traditional Chinese Medicine, Jiangxi University of Chinese Medicine, Nanchang, Jiangxi, China; ^4^ The Affiliated Stomatological Hospital, Jiangxi Medical College, Nanchang University, Nanchang, Jiangxi, China; ^5^ Jiangxi Provincial Clinical Research Center for Oral Diseases, Nanchang, Jiangxi, China

**Keywords:** oral squamous cell carcinoma, Brucea javanica oil, MTFR2, SOD2/H2O2, treatment

## Abstract

**Introduction:**

Oral squamous cell carcinoma (OSCC) is one of the most common malignant tumors in oral and maxillofacial region. The development of new chemotherapy agents and new drug combinations may improve patient survival and quality of life, but both surgery and radiotherapy have significant functional side effects and drug resistance, ultimately resulting in a 5-year survival rate of no more than 60% for OSCC patients. Studies have shown that Brucea javanica oil (BJO) extracts have anti-cancer effects against a variety of cancers, but little research has been reported on OSCC.

**Methods:**

CCK8, Colony formation, Scratch test and Transwell invasion assays were applied to determine the effects of BJO on the proliferation, migration, and invasion ability of OSCC cells in vitro. MTFR2 knockdown (shRNA) and overexpression (cDNA) OSCC cells were constructed to evaluate the effect of MTFR2 on the proliferation and invasion of OSCC cells. The nude mouse model of subcutaneous xenograft tumor was used to evaluate the effect of BJO on OSCC cells *in vivo*. PCR, western blot and immunohistochemistry were used to verify the expression of MTFR2, glycolysis markers and related pathway molecules after BJO treatment.

**Results:**

*In vivo* experiments using nude mice with xenografted OSCC cells and *in vitro* experiments with OSCC cell lines demonstrated that BJO treatment significantly inhibited the proliferation, migration, and invasiveness of OSCC cells. WB and PCR proved that BJO could effectively reduce the expression levels of MTFR2 and SOD2/H2O2 related signal transduction pathways. At the same time, the expression of oxidative phosphorylation markers increased, the expression of glycolytic markers decreased, and glycolysis-mediated decomposition of reactive oxygen species decreased, and H2O2 and oxygen levels decreased.In addition, when MTFR2 expression increased or decreased, SOD2/H2O2 expression also increased or decreased.

**Discussion:**

In this study, we concluded through in vitro and in vivo experiments that BJO may affect the SOD2/H2O2 signaling pathway by down-regulating MTFR2-mediated aerobic glycolysis, thereby inhibiting cell proliferation, Migration, and Invasion. The elucidation of this mechanism helps us to understand the molecular mechanism ofinhibiting OSCC invasion and metastasis by BJO, which has important clinical value or improving the survival rate of OSCC patients.

## 1 Introduction

Oral squamous cell carcinoma (OSCC) is one of the most common malignant tumors of the oral and maxillofacial region. Despite advances in the treatment of oral squamous cell carcinoma, the 5-year survival rate for OSCC patients does not exceed 60% because of tumor metastasis and subsequent recurrence (1). The development of new chemotherapeutic agents and new drug combinations may improve patient survival and quality of life, but both surgery and radiation therapy have significant functional side effects and drug resistance (2). As a result of the overuse of tobacco, alcohol, betel nut, and other substances in recent years, the incidence and mortality of oral cancer have increased in various regions of the world.

Traditional Chinese medicines are characterized by multi-targeting, low side effects and good effects, and have shown good anti-tumor effects in clinical practice. Brucea javanica belongs to the family Quasisteraceae. Its medicinal part is its dried and ripe fruit, and Brucea javanica oil (BJO) extracts from its body have been proven to have anti-cancer effects on various cancers (3, 4),including lung cancer (5), esophageal cancer (6), and ovarian cancer (7), but little research has been done on OSCC. Previous studies have demonstrated that oleic acid, a major component of Brucea javanica oil, can inhibit the proliferation and induce apoptosis and autophagy in oral squamous cell carcinoma cell lines (8); SOD2 is highly expressed in oral squamous cell carcinoma tissues (9); and the key Glycolysis enzymes HK2 and PKM2 can enhance the invasive metastasis of oral squamous cell carcinoma by up-regulating SOD2/H2O2 signaling pathway (10, 11). Mitochondrial fission regulator 2 (MTFR2) belongs to the MTFR1 family, plays an important role in the regulation of oxidative phosphorylation and is also involved in cancer carcinogenesis and progression (12). Lu G et al. reported that in breast cancer cell glucose metabolism, MTFR2 can convert oxidative phosphorylation to glycolysis for energy conversion by regulating HIF1αand HIF2α (13), suggesting that MTFR2 is involved in tumor cell glycolysis. Tumor cells can produce large amounts of ROS during glycolysis, and SOD2 can break down ROS into H2O2 and oxygen.

Therefore, we hypothesized that MTFR2 may regulate the SOD2/H2O2 signaling pathway by mediating aerobic glycolysis. Wang D et al. suggested that Brucea javanica oil induces cell cycle arrest and apoptosis in lung cancer cells through reactive oxygen species (ROS)-mediated mitochondrial dysfunction (14). Pan P et al. reported that Brucea javanica oil enhances the radio-sensitization of esophageal cancer cells by inhibiting HIF1α (15). Whereas MTFR2 plays an important function in mitochondria and glycolysis, MTFR2 can be oxidatively phosphorylated into glycolysis for energy conversion in breast cancer cell glucose metabolism. Therefore, we hypothesized that Brucea javanica oil may be able to regulate MTFR2.

**Figure 1:**
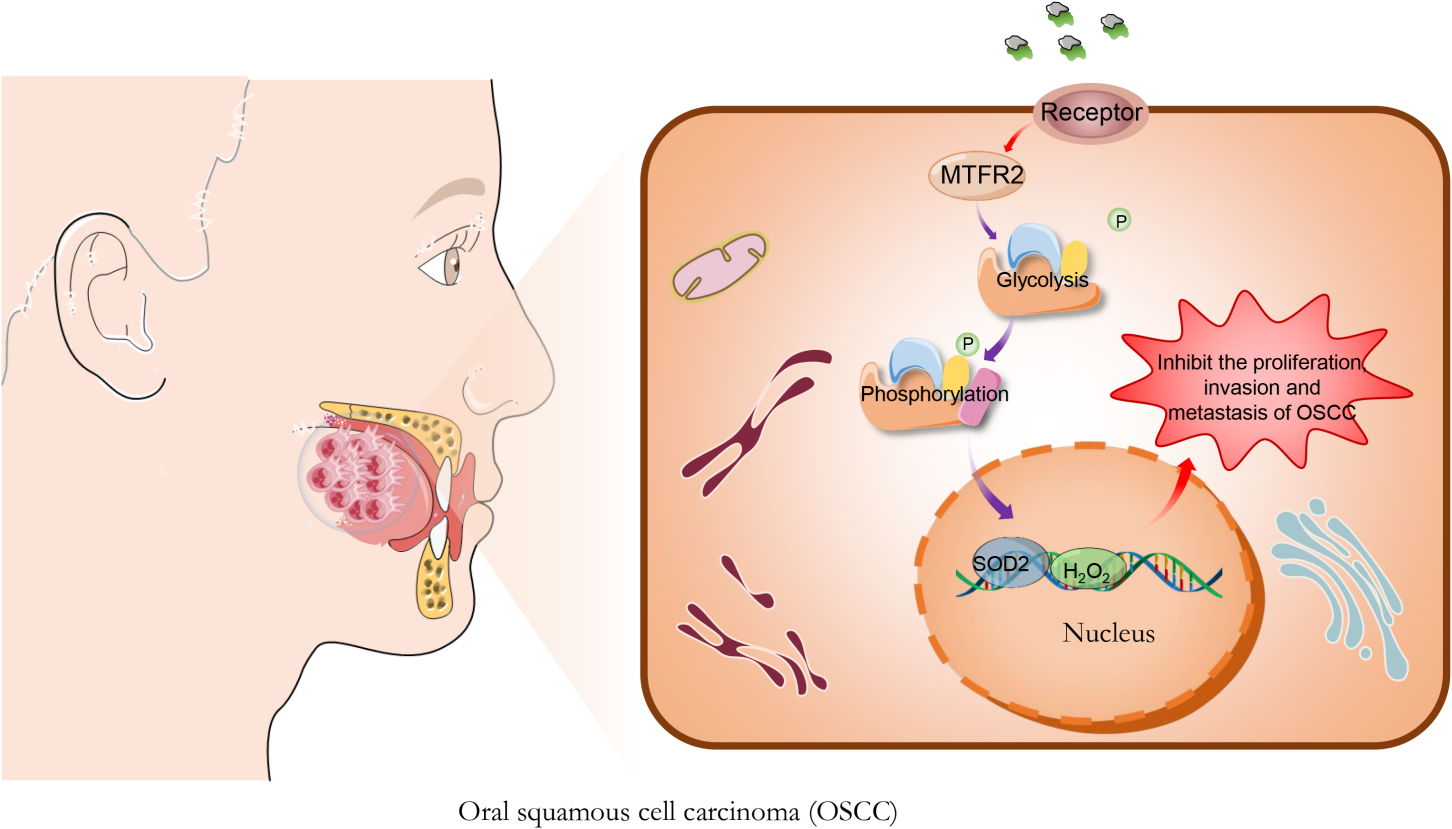
Brucea javanica oil (BJO) inhibits the proliferation of Oral squamous cell carcinoma. BJO may affect the SOD2/H2O2 signaling pathway through the down-regulation of MTFR2-mediated aerobic glycolysis, and thus inhibit the proliferation, invasion and metastasis of OSCC.

On the basis of the above analysis and the previous research of our group, we deduced from the *in vitro* and *in vivo* experiments that Brucea javanica oil may affect the SOD2/H2O2 signaling pathway through the down-regulation of MTFR2-mediated aerobic glycolysis, and thus inhibit the proliferation, invasion and metastasis of OSCC (**Figure 1**). The elucidation of this mechanism contributes to our understanding of the molecular mechanism of the inhibition of invasion and metastasis of OSCC by Brucea javanica oil, which is of great clinical value for improving the survival rate of patients with OSCC.

## 2 Materials and methods

### 2.1 Cell culture

Oral squamous carcinoma cell lines (UM1, CAL27) were selected, and the cells were routinely cultured in RPMI-1640 medium containing 10% FBS, placed in a saturated humidity incubator at a constant temperature of 37 °C and 5% CO2, and then digested and passaged with 0.25% EDTA-containing trypsin when the cells were spread over 80% of the field of view under a mirror. All the cells were routinely passaged twice after resuscitation, and then the above oral squamous carcinoma cell lines were divided into three groups, PBS (blank control group), Brucea javanica oil group (experimental group), and cisplatin (positive control group) group. The corresponding drug treatments were added, and the cells were subsequently in the incubator for 24 h for the subsequent experiments.

### 2.2 CCK-8 experiment

First, cells 100 μL (about 5000 ~ 10000 cells) were added to each hole of the 96-well plate, and the cells were cultured in a 5%CO2 cell incubator at 37°C for 24 h. Then add 10 μL of different concentrations of drug stimulation to each well. The 96-well plates were incubated in a cell incubator containing 5% CO2 air and 100% humidity at 37°C for an appropriate time. Add 10 μL of CCK-8 solution to each well. Incubate in a 5% CO2 incubator at 37°C for 1~4 h. Finally, the absorbance at 450 nm was measured by enzyme-labeled instrument. The experiment was repeated 3 times, cell inhibition rate (%) = (1 - average OD value of the treatment group/average OD value of the blank control group) ×100%.

### 2.3 Clone formation assay

After digestion of pancreatic enzymes of cells in logarithmic growth phase, the cell blast suspension was re-suspended in complete medium (basic medium +10% fetal bovine serum) and counted. Each experimental group was inoculated with 600 cells/Wells in 6-well plates and continued to culture for 14 days. After cloning, the cells were washed once with PBS, and fixed with 1 mL 4% paraformaldehyde per well for 30-60 min, and washed once with PBS. Add 1ml crystal violet dye solution to each well and stain cells for 10-20 min. The cells were washed several times with PBS, dried, and photographed by digital camera (the entire six-well plate and each well were photographed separately).Then manually calculate the cell colonies and draw a statistical bar chart using Prism 6 software.

### 2.4 Scratch test detection

First, 50-70% cells or 100-170w cells are planted in a 6-well plate. When the cell fusion rate reached 90%, the lid of the 6-well plate was lifted in a sterile environment, and the plate was scratched at the appropriate position. Without removing the medium, use a sterile 10μl pipette suction and, guided by a sterile straight edge, draw two parallel lines on plates about 5mm apart and 30mm long. According to the drug treatment time, reasonable change of medium. Finally, the camera was shot under the light mirror. The image of wound were obtained under a microscope (Nikon) at 100× magnification. The ImageJ program (version 1.53, National Institutes of Health, USA) was used to analyze images of *in vitro* scratch wound healing assays. The wound closure percentage was calculated based on the scratch width: Scratch width = Scratch area/scratch length; Wound closure percentage = (T0 hour scratch width - T24 hour scratch width)/T0 hour scratch width) ×100%.

### 2.5 Transwell invasion assay

The Transwell was rewarmed, the cells were digested and resuspended, the cell concentration was adjusted, 200μL of cell suspension was added to the chamber, and 600μL of medium containing 10% FBS was added to the lower part of the chamber. After 24 h of culture, the unmigrated cells in the chamber were gently wiped off with a cotton swab, the cells were fixed with 4% paraformaldehyde for 10min, stained with DAPI for 10 min, washed with single-steam water and air-dried, and then observed under an inverted microscope, and the number of cells passed through the chamber was determined by taking the upper, middle, lower, left and right five fields of view under a 200 x field of view, and the experiments were repeated three times for each group of cells.

### 2.6 Western blot experiment

For the western blot experiments, the cell lysates were collected after cell culture and centrifuged at 4°C, 14000r/min for 10 min. The supernatant was collected, the protein concentration was measured by BCA method, and electrophoresis was carried out in a gel electrophoresis tank by adding SDS-polyacrylamide at 10μL per lane and transferring it to the PVDF membrane; after being closed with skimmed milk powder at room temperature for 1 h, the primary antibody (MTFR2, SDHA, Uqcrfs1, Cyt-c, Fech, Glut2, LDHB, Actin, CDH1, Vimnetin, Snail, Slug, PCNA)was added and incubated at 4°C overnight, and the secondary antibody was incubated at room temperature for 2 h, chemiluminescent and scanned. Bands were semi-quantified by densitometrical analyses using ImageJ software.

MTFR2(1:500,MA5-27461), Snail(1:1000,14-9859-82), Slug(1:1000, MA5- 26385), PCNA(1:10000,13-3900), Cyt-c(1:1000,33-8500), Fech (1:500, PA5 -106355)antibodies were purchased from ThermoFisher. LDHB(1:1000, 3582S), Uqcrfs1(1:1000, 95231S)antibodies were purchased from Cell Signaling Technology. SDHA (1:10000, Ab14715) antibodies were purchased from Abcam. β-Actin (1:20000,HA722023),CDH1(1:5000,ET1607-75),Vimentin(1:20000,ET1610-39),Glut2(1:1000, ET1701-34) antibodies were purchased from HUABIO (**Table 1**).

**Table 1:** Related information about primary antibodies.

Catalog Number	Product Name	Species Reactivity	Dilution (cell/tissue)	Molecular weight	Source
HA722023	β-Actin	Rabbit	1:20000	42 kDa	HUABIO, China
MA5-27461	MTFR2	Mouse	1:500	43 KDa	ThermoFisher, America
14-9859-82	Snail	Mouse	1:1000	29 kDa	ThermoFisher, America
MA5-26385	Slug	Mouse	1:1000	30 KDa	ThermoFisher, America
13-3900	PCNA	Mouse	1:10000	36 KDa	ThermoFisher, America
ET1607-75	CDH1	Rabbit	1:5000	97 KDa	HUABIO, China
ET1610-39	Vimentin	Rabbit	1:20000	54 KDa	HUABIO, China
Ab14715	SDHA	Mouse	1:10000	70 KDa	Abcam, Britain
ET1701-34	Glut2	Rabbit	1:1000	57 KDa	HUABIO, China
33-8500	Cyt-c	Mouse	1:1000	15 KDa	ThermoFisher, America
3582S	LDHB	Rabbit	1:1000	37 KDa	Cell Signaling Technology, America
PA5-106355	Fech	Rabbit	1:500	48 KDa	ThermoFisher, America
95231S	Uqcrfs1	Rabbit	1:1000	23 KDa	Cell Signaling Technology, America

### 2.7 TSCC xenografts in nude mice

To investigate the anticancer effect of BJO *in vivo*, CAL27 cells (1x10^7^/0.2 mL) were injected subcutaneously into 4-6 week-old female BALB/c nude mice (purchased from Beijing Weitong Lihua Laboratory Animal Technology Co., Ltd.) subcutaneously on the lower dorsal side of the right upper limb. After 8 days, the maximum diameter of each xenograft tumor in all groups was greater than 5 mm. The mice were then randomly divided into 3 groups (the control group, the BJO treated group, and the CIS treated group, n = 5/group). The control group (normal saline 200 mg/kg), the BJO group (BJO 200 mg/kg), and the CIS group (CIS 200 mg/kg) were administered intraperitoneally every three days for a total of 12 times. In addition, the animals were euthanized on the 45th day. Tumors were measured on days 15, 19, 23, 27 and 35, with a standard caliper and the tumor volumes were calculated as 0.5×length×width^2^, and a tumor growth curve (y=Aekday) was obtained as previously described. At the end of the experiments, the tumors were weighed after being separated from the surrounding muscles and dermis.

The tumor samples were fixed in 4% paraformaldehyde and embedded in paraffin wax. Sample sections were dewaxed, rehydrated and stained with Mayer’s hematoxylin and eosin Y solution. Immunohistochemistry (IHC) was used to detect the expression of MTFR2 in xenograft sections.

### 2.8 Statistical analysis

The results are presented as means ± standard deviations (SDs), for which at least three samples were used. One-way analysis of variance (ANOVA) and Bonferroni-multiple comparisons were used to analyze the data. All analyses were performed using GraphPad Prism 9.0, SPSS and Origin 2019b. P values < 0.05 indicate statistical significance. ns in the graphs indicates no significance; * p < 0.05, ** p < 0.01, and *** p < 0.001.

## 3 Results

### 3.1 Brucea javanica oil inhibits the proliferation, invasion and migration of OSCC

**Figure 2:**
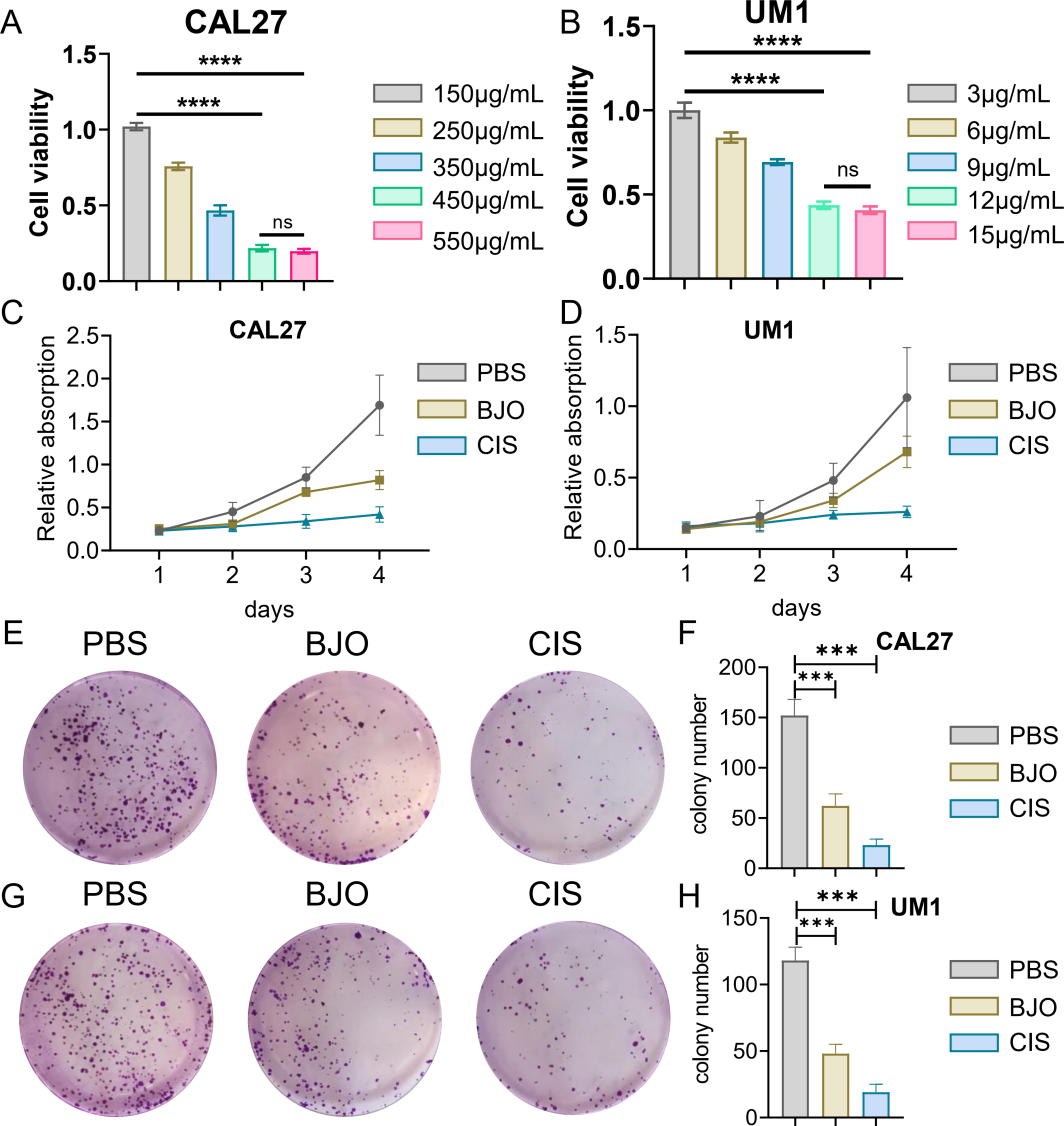
BJO can inhibit the proliferation of Oral squamous cell carcinoma (CAL27, UM1). **(A, B)** CCK-8 experiment of BJO and CIS with different drug concentrations. **(C, D)** PBS, BJO and CIS were added to detect the relative absorbance values of CAL27 and UM1 by CCK-8 assay. **(E–H)** The number of clones formed by clone formation assay was detected. (***P < 0.001, ****P<0.0001). "ns" indicates no significant difference between the two sides.

**Figure 3:**
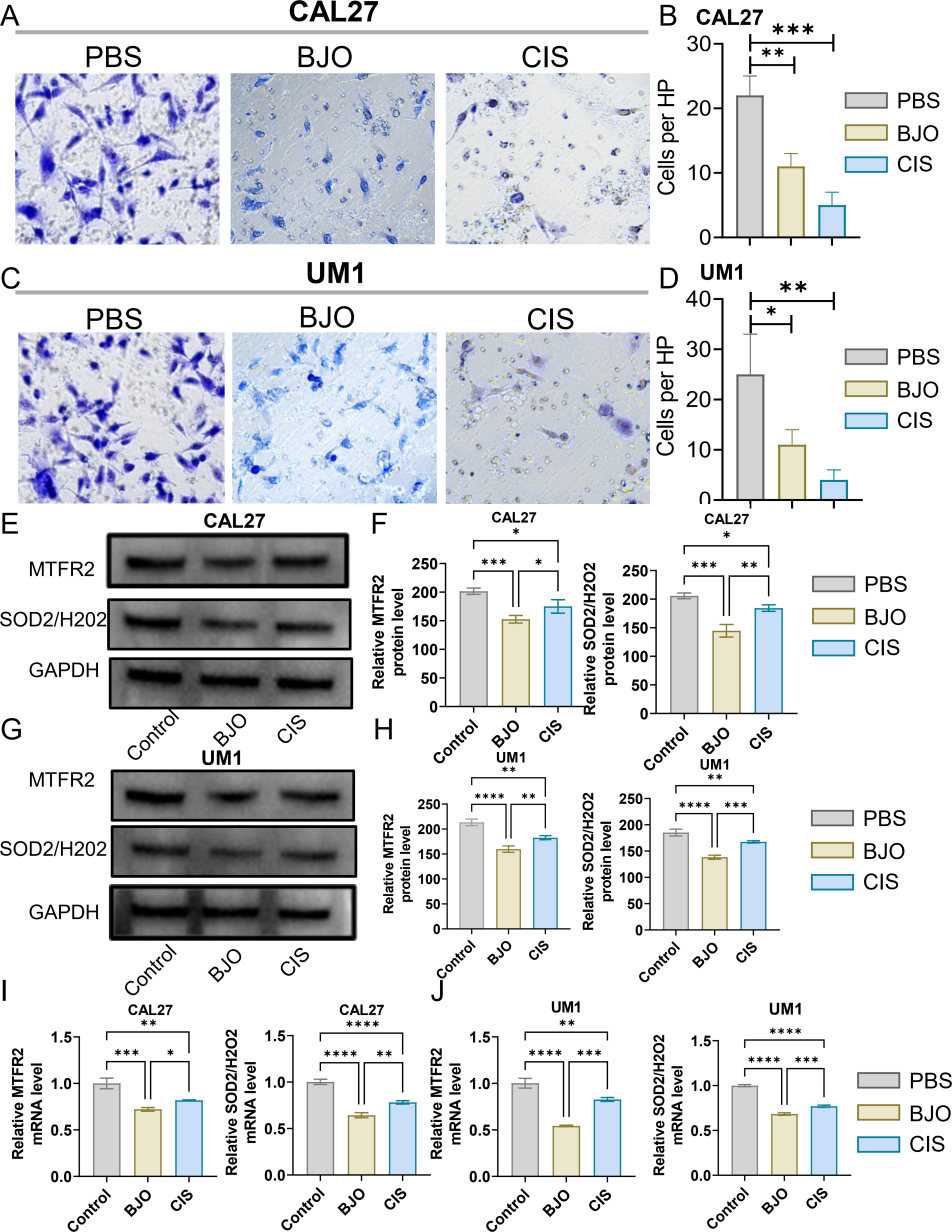
BJO can inhibit the invasion of Oral squamous cell carcinoma (CAL27, UM1). **(A–D)** PBS, BJO and CIS were added to detect the invasion ability of CAL27 and UM1 by Transwell invasion assay. **(E–H)** The expression of MTFR2, SOD2/H2O2 in CAL27 and UM1 was detected by WB assay. **(I, J)** The expression of MTFR2, SOD2/H2O2 in CAL27 and UM1 was detected by PCR assay. (*P < 0.05, **P < 0.01,***P < 0.001,****P<0.0001).

**Figure 4:**
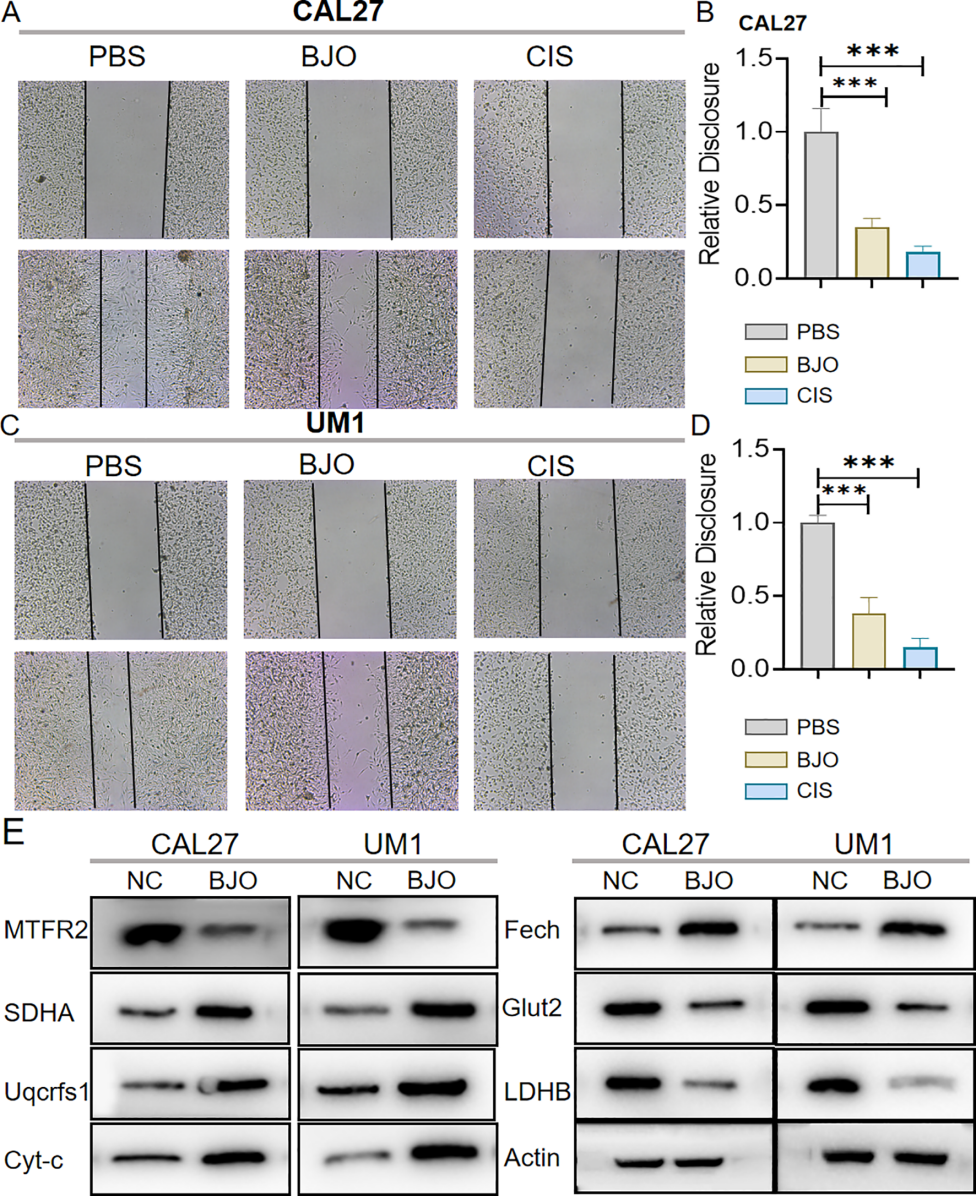
BJO can inhibit the migration of Oral squamous cell carcinoma (CAL27, UM1). **(A–D)** PBS, BJO and CIS were added to detect the migration ability of CAL27 and UM1 by scratch assay. **(E)** Western Blot was used to detect the changes of MTFR2 expression, oxidative phosphorylation markers (Cyt-c, SDHA, Fech, Uqcrfs1, etc.) and glycolysis markers (LDHB, Glut2, etc.) after treatment with BJO. (***P < 0.001).

**Figure 5:**
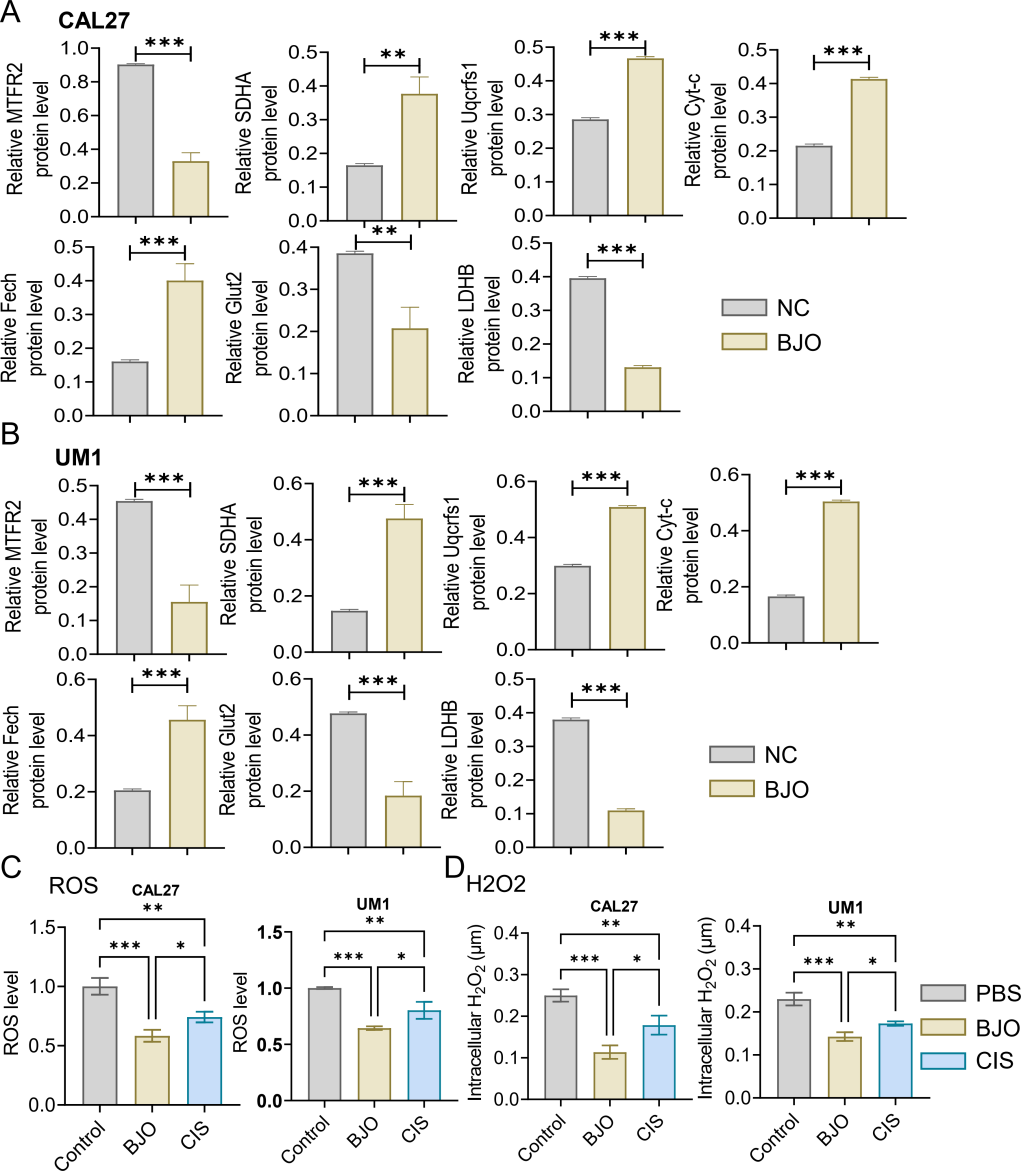
Expression analysis of MTFR2, oxidative phosphorylation markers, glycolysis markers, ROS and H₂O₂ (CAL27, UM1). **(A, B)** The MTFR2 expression, oxidative phosphorylation markers (Cyt-c, SDHA, Fech, Uqcrfs1, etc.) and glycolysis markers (LDHB, Glut2, etc.) quantitative analysis. **(C, D)** PBS, BJO and CIS were added to detect the ROS, H2O2 expression of CAL27 and UM1. (*P < 0.05, **P < 0.01 and ***P < 0.001).

We used the CCK-8 assay and clony formation assay to test the effect of Brucea javanica oil on the proliferation of oral squamous carcinoma. First, we explored the effective concentrations of BJO and CIS, and found that the optimal effective concentrations of BJO and CIS were 450μg/mL and 12μg/mL, respectively (**Figures 2A, B**). Then, PBS, BJO and CIS were added to tongue squamous cell (CAL27, UM1) culture dishes to detect relative absorbance (**Figures 2C, D**). Then their clone formation numbers were counted (**Figures 2E-H**). A transwell invasion assay was used to test the invasive ability of Brucea javanica oil on CAL27 and UM1 cells (**Figures 3A-D**), and the number of cells in each well was counted. WB (**Figures 3E-H**) and PCR (**Figures 3I, J**) proved that BJO could effectively reduce the expression levels of MTFR2 and SOD2/H2O2 related signal transduction pathways. The scratch test was used to detect the migration ability of Brucea javanica oil on oral squamous carcinoma, and the relative exposure was calculated (**Figures 4A-D**). It can be found that each of the above experimental groups had reduced values compared to the control group. Western Blotting was used to detect the decrease in the expression of MTFR2, increases in the expression of oxidative phosphorylation markers (Cyt c, SDHA, Fech, Uqcrfs1, etc.), and decreases in the expression of glycolysis markers (LDHB, Glut2, etc.) after treatment with Brucea javanica oil. Actin was used as an internal reference (**Figures 4E**, **5A, B**). At the same time, glycolytic-mediated ROS breakdown was reduced, and H2O2 and oxygen levels were reduced (**Figures 5C, D**).

### 3.2 Effects of MTFR2 knockdown or overexpression on the proliferation invasion and metastasis of OSCC

**Figure 6:**
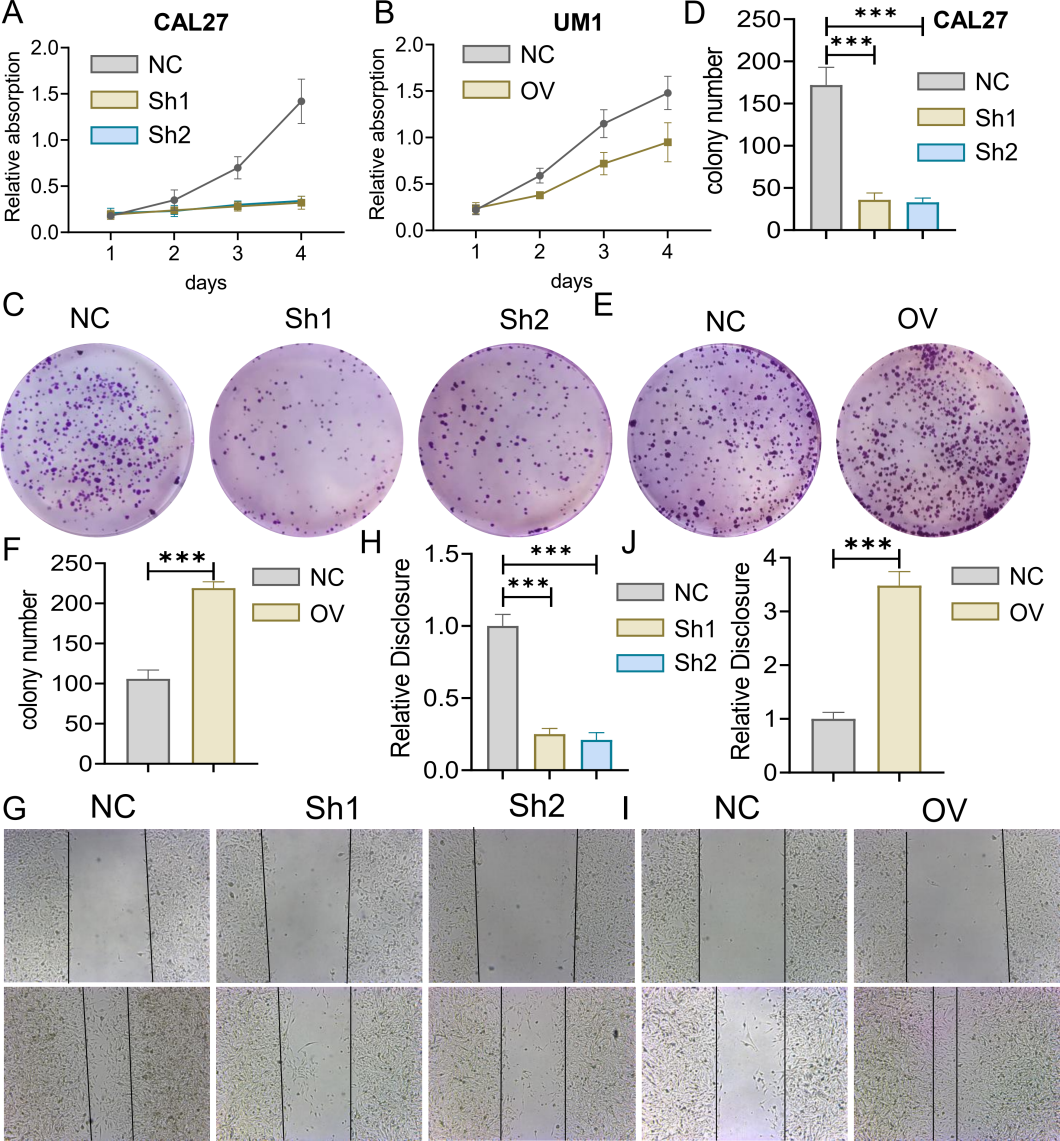
Effect of MTFR2 gene knockout or overexpression on the proliferation of oral squamous cell carcinoma cells. MTFR2 gene knockout (shRNA) or overexpression (cDNA) of oral squamous cell carcinoma stable metastases were constructed. **(A, B)** CCK-8 assay was used to detect cell viability. **(C-F)** Cell proliferation was detected by clonal assay. **(G-J)** Cell migration ability was detected by scratch assay. (***P < 0.001).

**Figure 7:**
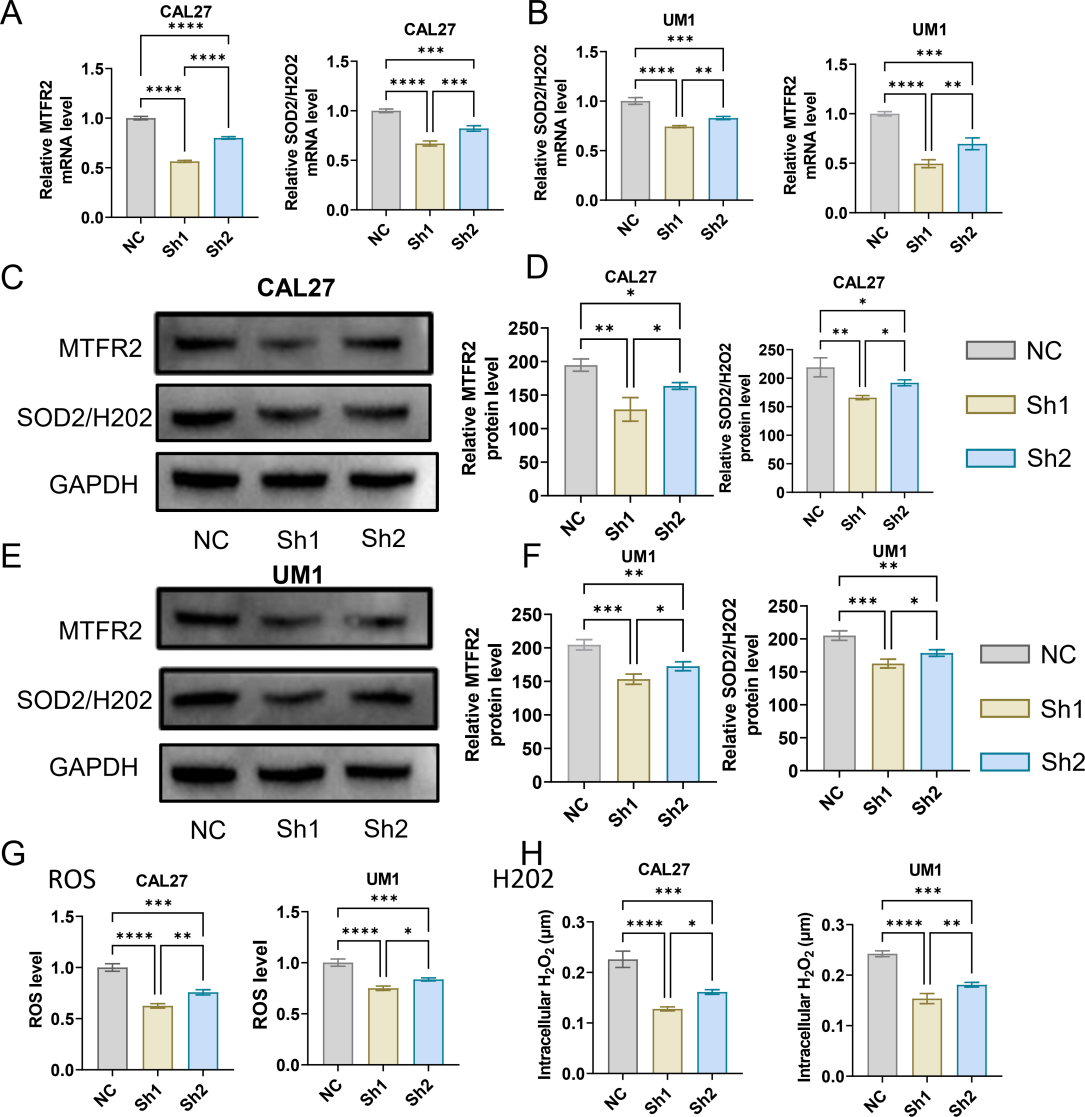
Studies on the regulatory relationship between knockdown of MTFR2 and SOD2/H2O2 signal (CAL27, UM1). **(A, B)** The expression of MTFR2, SOD2/H2O2 in CAL27 and UM1 was detected by PCR. **(C–F)** The expression of MTFR2, SOD2/H2O2 in CAL27 and UM1 was detected by WB. **(G, H)** The expression of ROS, H2O2 in CAL27 and UM1 was detected by ROS kit. (*P < 0.05, **P < 0.01,***P < 0.001,****P<0.0001).

**Figure 8:**
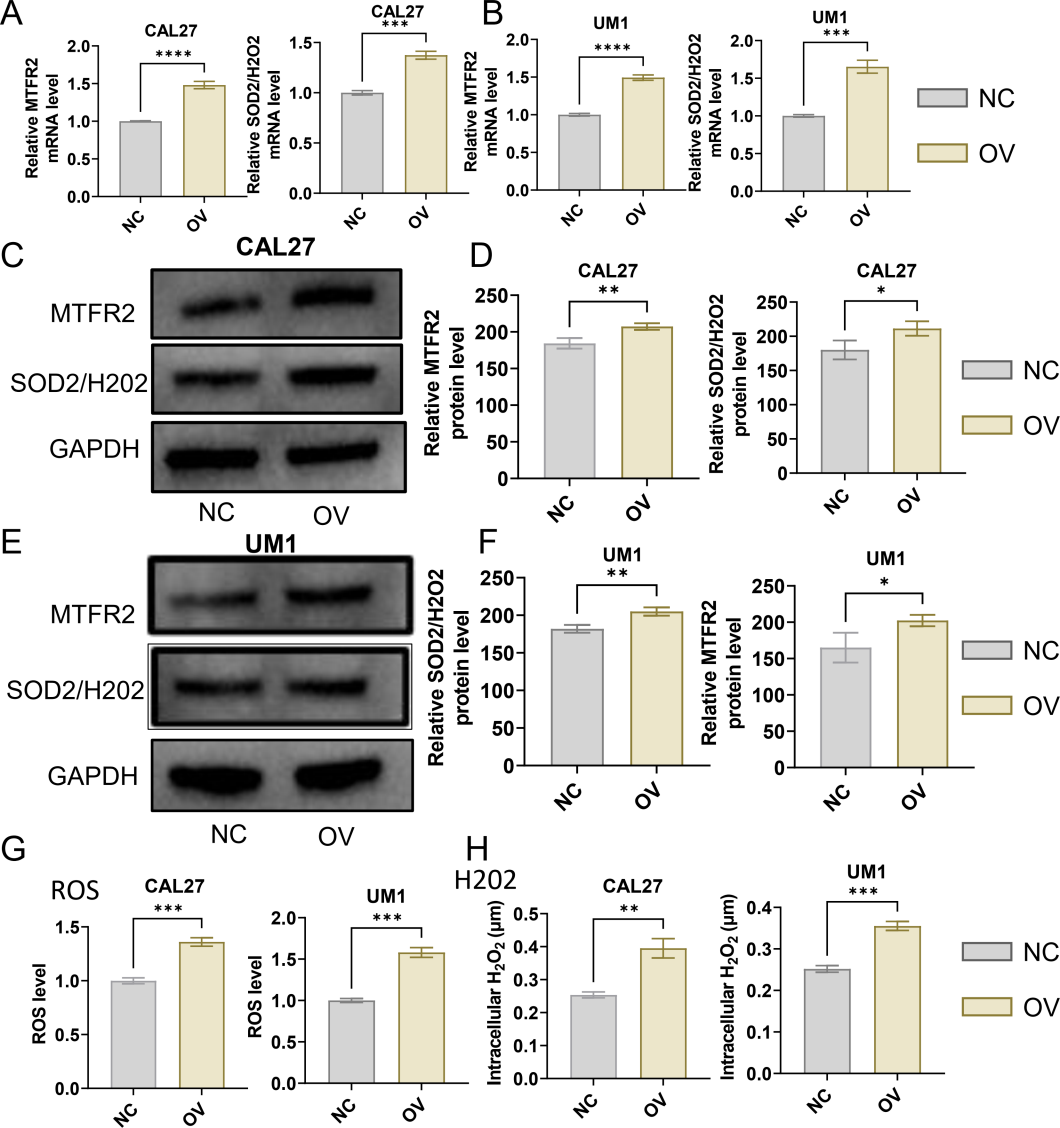
Studies on the regulatory relationship between overexpression of MTFR2 and SOD2/H2O2 signal (CAL27, UM1). **(A, B)** The expression of MTFR2, SOD2/H2O2 in CAL27 and UM1 was detected by PCR. **(C–F)** The expression of MTFR2, SOD2/H2O2 in CAL27 and UM1 was detected by WB. **(G, H)** The expression of ROS, H2O2 in CAL27 and UM1 was detected by ROS kit (*P < 0.05, **P < 0.01, ***P < 0.001, ****P<0.0001).

**Figure 9:**
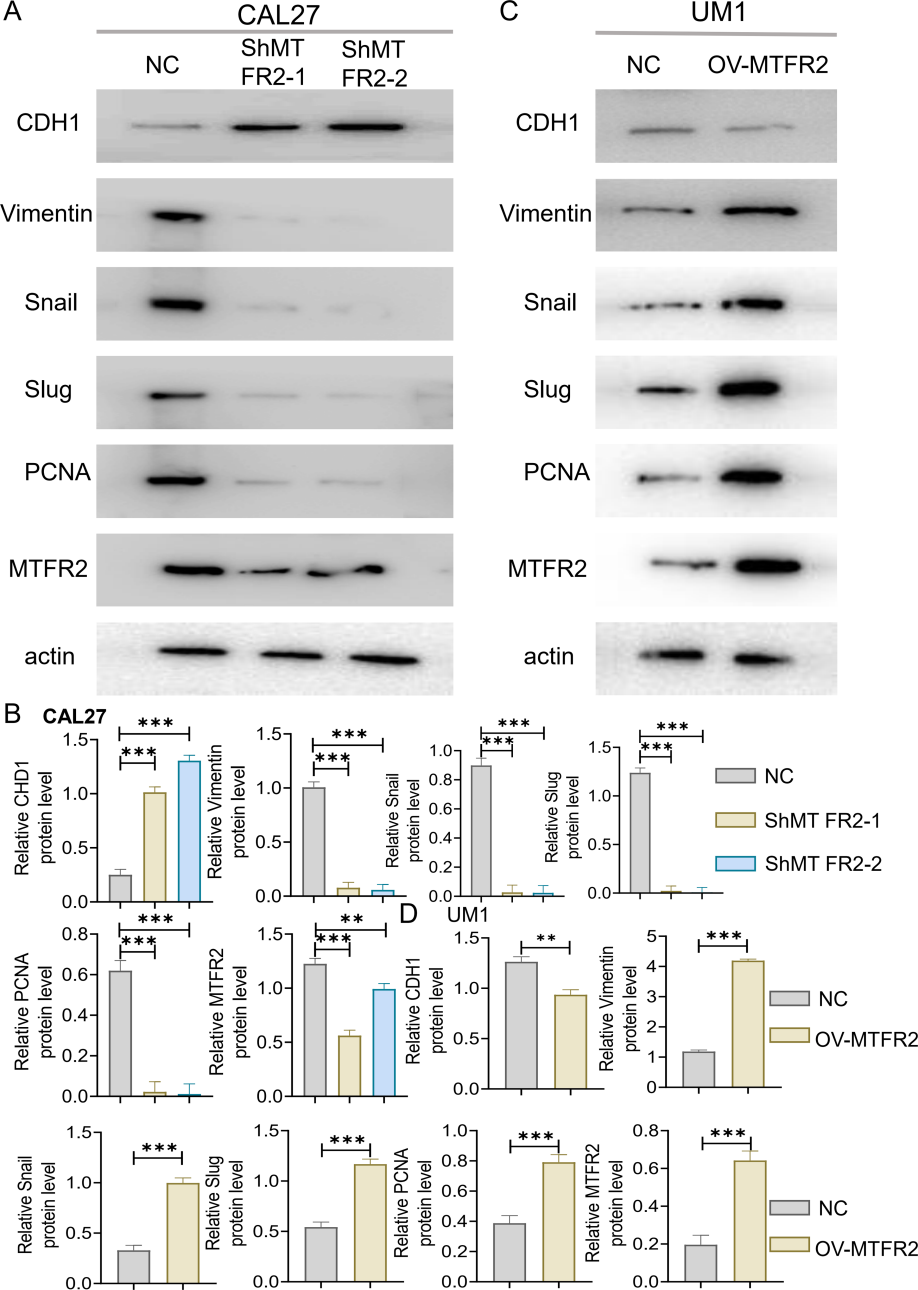
Effect of knockout or overexpression of MTFR2 gene on expression of related proteins. MTFR2 gene knockout (shRNA) or overexpression (cDNA) of oral squamous cell carcinoma stable metastases were constructed. **(A–D)** Western Blot analysis of CDH1, PCNA, MTFR2 and EMT-related proteins expression and its quantitative analysis. (*P < 0.05, **P < 0.01, ***P < 0.001 and ****P < 0.0001).

The MTFR2 knockdown (shRNA) and overexpression (cDNA) oral squamous carcinoma stable transplants were constructed, and the effects of MTFR2 knockdown or overexpression on the proliferation of oral squamous carcinoma cells were detected by using CCK-8 and clone formation assays, respectively (**Figures 6A-F**). Scratch assay was used to detect the effect of MTFR2 knockdown or overexpression on the migration ability of oral squamous carcinoma cells (**Figures 6G-J**). In order to further verify the relationship between MTFR2 expression and SOD2/H2O2 pathway, we also observed the expression of SOD2/H2O2 by knocking down MTFR2 and overexpressing it. The results showed that when MTFR2 expression increased or decreased, SOD2/H2O2 expression also increased or decreased by PCR (**Figures 7A, B**, **8A, B**) and WB (**Figures 7C-H**, **8C-H**) detection. Western Blotting was used to detect the effect of MTFR2 knockdown or overexpression on the proliferation, invasion and migration ability of oral squamous carcinoma cells. After knockdown the expression of CDH1 in CAL27 increased, decreased expression of Vimnetin, Snail, Slug, PCNA, MTFR2 in CAL27; CDH1 overexpression in CAL27 is decreased, Vimnetin, Snail, Slug, PCNA, MTFR2 overexpression rises in CAL27. Actin was used as an internal reference (**Figures 9A-D**).

### 3.3 BJO represses the xenograft tumor growth *in vivo*

**Figure 10:**
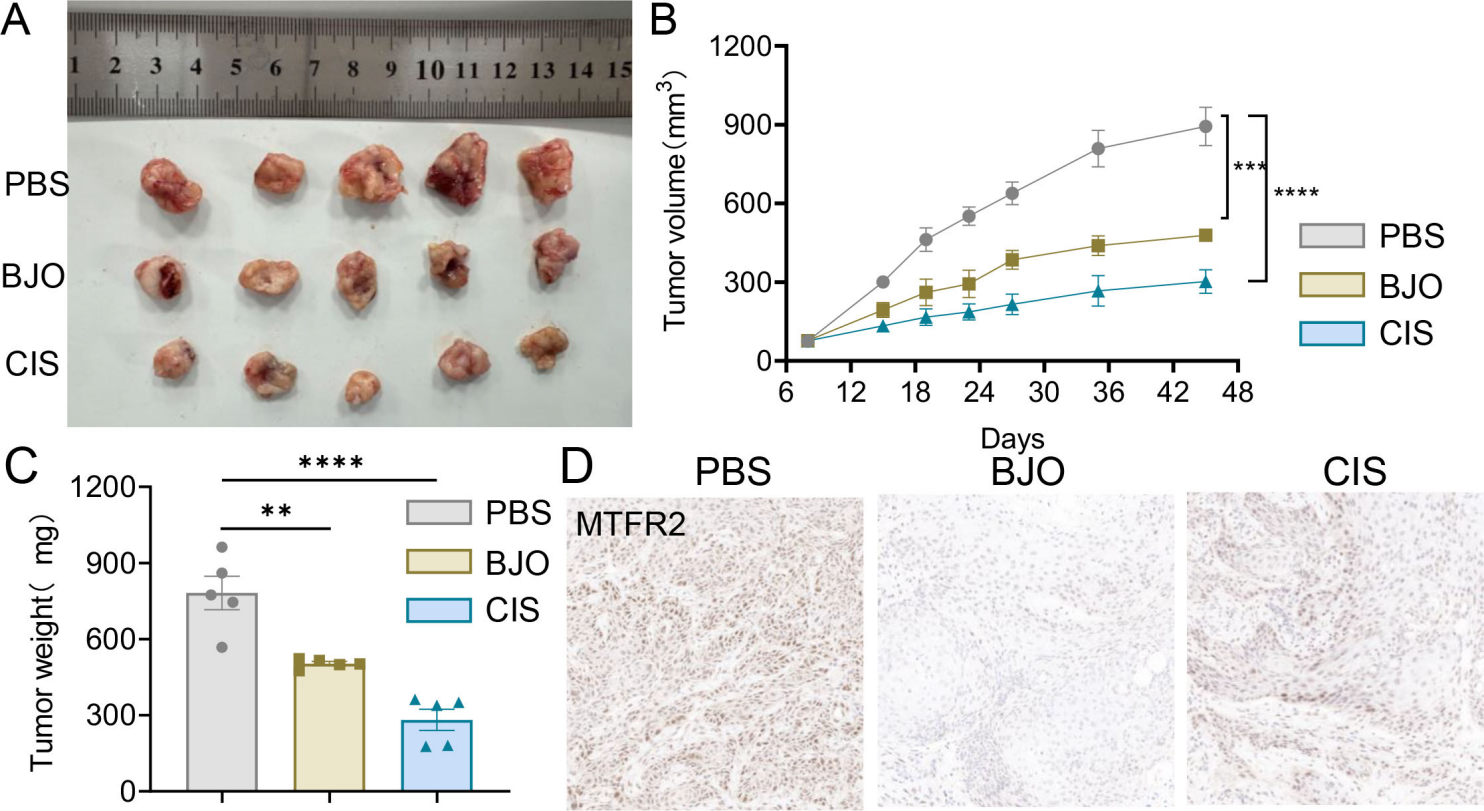
BJO inhibited the growth of CAL27 xenograft tumors in nude mice. **(A)** Representative photographs of the gross tumors from nude mice. **(B)** Graphs represent the average tumor volumes of CAL27 xenografts in mice from the control, BJO-treated groups and CIS-treated groups. **(C)** Graphs represent the average weights of tumors from the control, BJO-treated groups and CIS-treated groups. **(D)** The expression of MTFR2 was detected by immunohistochemical staining and the expression of MTFR2 was decreased after BJO and CIS treatment. (**P < 0.01, ***P < 0.001, ****P < 0.0001).

To investigate whether BJO inhibits the invasion and metastasis of TSCC *in vivo*, we chose the CAL27 cell line to establish xenograft tumors in nude mice. The mice were randomly assigned to three groups (Control, BJO, and CIS) when the tumor volume was 76 ± 10mm^3^. Compared with that in the control group, the tumor volume in the groups treated with BJO and CIS showed a markedly inhibited tumor growth compared with the control group. The tumor in the BJO and CIS groups were significantly smaller than that of control group (**Figure 10A**). The BJO group tumor inhibition rates on day 45 weas 46.42%, and the CIS group tumor inhibition rate on day 45 were 66.12% according to the tumor volume (**Figures 10B, C**). Immunohistochemical analyses also showed that BJO and CIS strongly inhibited the expression of MTFR2 expression in the xenograft tumors (**Figure 10D**).

## 4 Discussion

Brucea javanica oil has therapeutic effects on many cancers, and recent studies have shown that Brucea javanica oil can inhibit tongue squamous cell invasion and metastasis by modulating the miR/138/EZH2 pathway (16); However, its specific mechanism of action on OSCC has not been reported. In this study, we demonstrated that Brucea javanica oil could inhibit the proliferation, invasion and metastasis of oral squamous cell carcinoma by down-regulating MTFR2-mediated aerobic glycolysis and modulating the SOD2/H2O2 signaling pathway.

It is well known that cancer cells derive their metabolic energy from ATP produced by mitochondria through oxidative phosphorylation. And MTFR2 promotes mitochondrial division and is involved in cancer development. Some studies have reported that downregulation of MTFR2 inhibits the proliferation, invasion and migration of gastric cancer cells *in vitro* and *in vivo* (17). Lian Z et al. reported that MTFR2 can promote lung adenocarcinoma cell proliferation and metastasis through the AKT pathway, and it can be used as a prognostic biomarker for lung adenocarcinoma patients (12). We used Western Blotting to detect the decreased expression of MTFR2 after treatment with Brucea javanica oil; therefore, we hypothesized that Brucea javanica oil might be able to regulate MTFR2.

SOD2 is involved in tumor progression through a variety of mechanisms (18). It has been found that SOD2 is highly expressed in a variety of tumors such as oral squamous cell carcinoma cells and gastric cancer cells, and plays an important role in several biological aspects of tumors (9, 17). Wang Y et al. concluded that overexpression of SOD2 abrogated the antitumor effect of ZNF-148 deficiency on the proliferation and pyrokinesis of breast cancer cells (19).

In conclusion, through *in vitro* and *in vivo* experiments, the study revealed that Brucea javanica oil may affect the SOD2/H2O2 signaling pathway through the down-regulation of MTFR2-mediated aerobic glycolysis, and thus inhibiting the proliferation and invasion metastasis of OSCC.

## Data Availability

The raw data supporting the conclusions of this article will be made available by the authors, without undue reservation.
